# Dietary Inflammatory Index and S-Klotho Plasma Levels in Middle-Aged Adults

**DOI:** 10.3390/nu12020281

**Published:** 2020-01-21

**Authors:** Lucas Jurado-Fasoli, Manuel J. Castillo, Francisco J. Amaro-Gahete

**Affiliations:** Department of Physiology, Faculty of Medicine, University of Granada, 18016 Granada, Spain; mcgarzon@ugr.es (M.J.C.); amarof@ugr.es (F.J.A.-G.)

**Keywords:** aging, dietary inflammatory index, Klotho, inflammation

## Abstract

Background: Soluble Klotho (S-Klotho) is an aging suppressor with a close link with inflammation. However, it is still unknown whether the dietary inflammatory potential is associated with S-Klotho plasma level. We aimed to investigate the association of the Dietary Inflammatory Index (DII) with S-Klotho plasma levels in middle-aged sedentary adults. Methods: 73 middle-aged sedentary adults (40–65 years old) participated in the present study. DII was determined from 28 dietary items obtained by 24 h recalls and food frequency questionnaires. The S-Klotho plasma levels were measured using a solid-phase sandwich enzyme-linked immunosorbent assay. Results: a weak positive association was observed between DII and S-Klotho plasma levels (β = 52.223, R^2^ = 0.057, *p* = 0.043), which disappeared after controlling for body mass index (*p* = 0.057). Conclusions: A pro-inflammatory dietary pattern measured with the DII was slightly and positively associated with S-Klotho plasma levels in middle-aged sedentary adults.

## 1. Introduction

Human life expectancy is projected to increase at least 65% for women and 85% for men in the following years in developed countries [1]. Parallelly, the rise of non-communicable chronic disease prevalence has increased health care costs [1]. In this sense, previous evidence suggests a close link between chronic inflammation during the aging process and the incidence of several age-associated chronic diseases in humans (i.e., cardiovascular diseases, diabetes mellitus, cancer, inflammatory bowel disease, and asthma, among others) [2,3].

Chronic inflammation may be favored by several factors such as age, obesity, smoking, stress, sleep disorders and/or diet [4]. Indeed, previous studies have consistently demonstrated that several dietary factors influence chronic inflammation [5,6,7]. Diets with high omega-3 intake, low saturated and trans fatty acids intake, and high intake of fruits, vegetables, nuts and whole grains have been associated with a lower generation of inflammation [8]. Recently, the Dietary Inflammatory Index (DII) has been developed, a dietary tool which determines the inflammatory potential of an specific individual diet [9]. The DII has been originally validated with several inflammatory biomarkers by Shivappa et al. [9], and later validated in different populations with inflammatory biomarkers [10,11,12]. Therefore, a high DII (i.e., a pro-inflammatory diet) may be associated with increased risk of suffering chronic diseases and/or all-cause mortality in humans [7,13].

Klotho is a transmembrane protein with important anti-aging functions [14]. The shed form of the Klotho protein (S-Klotho) is produced by proteolytic cleavage from the membrane Klotho and can be found in blood, urine and cerebrospinal fluid [14,15,16]. S-Klotho exerts different metabolic functions including improvement of insulin sensitivity and glucose uptake and decreased oxidative stress and chronic inflammation [16,17]. It has been also related to a low risk of all-cause mortality (participants with <575 pg/mL had an increased risk of death) and cardiovascular disease (626 pg/mL in cardiovascular disease participants vs 671 pg/mL in those without a cardiovascular disease) in humans [18,19]. In this sense, there is a bidirectional relationship between S-Klotho and systemic inflammation: systemic inflammation downregulates S-Klotho plasma levels, and S-Klotho plasma levels downregulate systemic inflammation [20]. However, to the best of our knowledge, there is a lack of studies investigating the relationship between the inflammatory potential of the diet and S-Klotho plasma levels. It seems crucial to understand clearly whether a pro-inflammatory dietary pattern could modulate S-Klotho plasma levels in humans. Therefore, this study aimed to investigate the association of the DII with S-Klotho plasma levels in middle-aged sedentary adults.

## 2. Materials and Methods

### 2.1. Participants

The current study has a cross-sectional design from data collected at the baseline in the FIT-AGEING clinical trial [ClinicalTrials.gov. ID: NCT03334357] [21]. The study subjects were 73 middle-aged sedentary adults (38 women) aged 53.6 ± 5.2 years. All assessments were made at the Sport and Health University Research Institute (iMUDS) (iMUDS) in Granada (Spain) during September-October (2016 and 2017). All participants were non-physically active (<20 min of moderate-intensity physical activity on 3 days/week), had a stable weight (weight changes <5 kg) over the last 5 months, were non-habitual smokers, and were healthy. The study was performed in accordance with the last revised Declaration of Helsinki (2013 revision) and was approved by the Ethics Committee on Human Research of the University of Granada (CEI-Granada) (0838-N-2017). All participants gave their written informed consent to be included.

### 2.2. Procedures

#### 2.2.1. Dietary Data

Diet was assessed by qualified and well-trained dietitians using three 24 h recalls undertaken in non-consecutive days (i.e., 2 weekdays and 1 weekend day) and a previously validated food frequency questionnaire (FFQ) [22].

The participants were asked to recall all food consumed during the previous day. The interviews involved a detailed assessment and description of the food consumption using different colored photographs of different portion sizes of food to improve the participants’ accuracy of food quantification [23]. The data registered by the 24 h recalls were introduced in the EvalFINUT^®^ software (FINUT, Granada, Spain), which is based on the USDA (United States Department of Agriculture) and BEDCA (“Base de Datos Española de Composición de Alimentos”) databases. Energy, macronutrient, and micronutrient intake data were obtained by EvalFINUT^®^.

Food frequency consumption was assessed using a 100-food item FFQ. The participants were asked how often they had consumed each food item on average over the last three months. Dietitians insisted on ensuring that answers were related to usual dietary factors and not to recent dietary changes.

All questionnaires were performed during face-to-face interviews in a quiet, well-lit and spacious room.

#### 2.2.2. Dietary Inflammatory Index (DII)

The DII score describes the inflammatory properties of the diet [9]. The DII was originally developed using 45 parameters. However, it could also be calculated using >20 items from the last-mentioned list of parameters [9]. The calculation of the DII score was based on 28 nutrients and food components (i.e., energy, fat, carbohydrate, protein, fiber, alcohol, saturated fatty acids, monounsaturated fatty acids, polyunsaturated fatty acids, cholesterol, β-carotene, vitamin C, vitamin D, vitamin E, niacin, thiamin, riboflavin, vitamin B6, vitamin B12, folate, iron, magnesium, selenium, zinc, garlic, onion, tea and pepper). We subtracted the global average intake (obtained from Shivappa et al. [9]) from the dietary amount reported by the participant and we divided it by the standard deviation of the global average intake. This value was transformed to a centered percentile score to minimize the effect of ‘right skewing’. Then, every percentile score of every food parameter was multiplied by its respective inflammatory effect score (obtained from Shivappa et al. [9]). These new values were then summed to obtain the overall DII score of each participant. The DII score characterizes the inflammatory properties of the participant’s diet from anti-inflammatory (low DII scores) to pro-inflammatory (high DII scores).

#### 2.2.3. S-Klotho Plasma Levels

Blood samples were collected from the antecubital vein in a supine position. All participants were requested to fast for 12 h, to abstain from drugs and/or caffeine, to eat an established dinner before sampling, and to not do any physical activity at moderate intensity (24 h before) and/or vigorous intensity (48 h before).

S-Klotho was determined according to a solid-phase sandwich enzyme-linked immunosorbent assay kit (Demeditec, Kiel, Germany) strictly following the manufacturer’s instruction. To determine intra- and inter-assay coefficients of variation, two different doses of purified S-Klotho were measured.

#### 2.2.4. Anthropometric Measurements

Weight was measured to the nearest 0.1 kg, and height to the nearest 0.1 cm using a Seca scale and a stadiometer (model 799, Electronic Column Scale, Hamburg, Germany) following the International Society for the Advancement of Kinanthropometry protocol [24]. Body mass index (BMI) was calculated from height and weight (kg/m^2^). We determined waist circumference at the midpoint between the iliac crest and the bottom of the rib cage after the end of a normal expiration. Hip was recorded as the maximum circumference over the buttocks. The waist-hip ratio was subsequently calculated.

#### 2.2.5. Blood Pressure Assessment

The blood pressure was measured with an automatic monitor (Omrom^®^ HEM 705 CP, Health-care Co, Kyoto, Japan) following the recommendations of the European Society of Cardiology (on the right arm, with the participants in a supine position, and after 10 min of rest) [25]. It was measured twice and the mean calculated.

### 2.3. Statistical Analyses

The present study is based on a secondary analysis using baseline data from the FIT-AGEING project, and therefore a specific power calculation was not developed for the present study [21].

The distribution of the variables was verified using the Shapiro–Wilk test, skewness and kurtosis values, visual check of histograms, Q-Q, and box plots. The descriptive parameters were reported as mean and standard deviation. We conducted the analyses in men and women together since there was no sex interaction. We conducted the curve estimation analyses to choose the regression analyses to use.

We built a simple linear regression model to test the association of the DII with S-Klotho plasma levels. In order to check whether DII predicts S-Klotho independently of potential confounders, a hierarchical regression analysis was performed taking into account the following outcomes: energy intake, sex, BMI, waist-hip ratio, medication use and systolic and diastolic blood pressure.

## 3. Results

Table 1 shows the baseline characteristics of the study participants.

**Figure 1:**
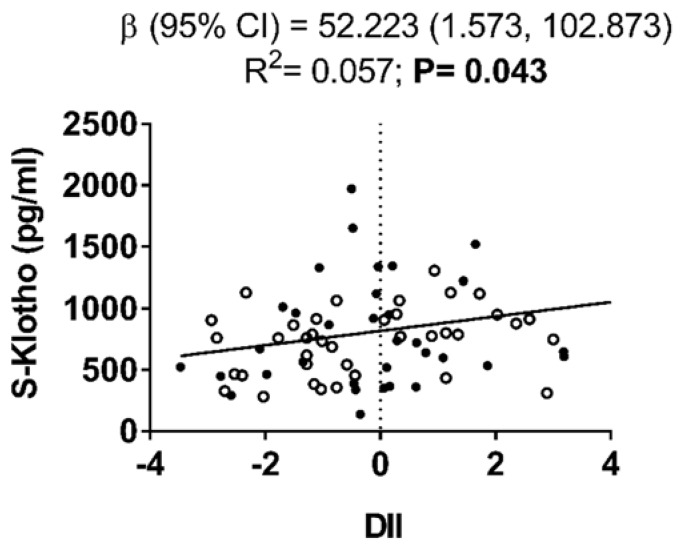
Association of dietary inflammatory index with S-Klotho plasma levels in young sedentary adults. Non-standardized β coefficient [95% confidence interval], R^2^ and *p* value are provided. Black and white dots represent men and women respectively.

We observed a positive association between DII and S-Klotho plasma levels (β = 52.223, R^2^ = 0.057, *p* = 0.043; Figure 1).

Based on this hierarchical regression, we decided to discard energy intake, sex, WHiR, medication use, systolic and diastolic blood pressure as confounders variables including only BMI as potential confounder (Table 2).

The association disappeared when the analysis was adjusted for BMI (β = 46.651 (−1.350, 94.653), R^2^ = 0.170, *p* = 0.057).

## 4. Discussion

The current study shows for the first time that DII is positively associated with S-Klotho plasma levels in middle-aged sedentary adults. However, this result should be interpreted with caution because the association was weak and disappeared after adjusting for BMI. Given that the beta coefficient was quite similar considering the raw and the adjusted model, respectively (i.e., 52.223 and 46.651, respectively), it could have a marked clinical significance.

S-Klotho has previously demonstrated to exert anti-inflammatory effects [20]. In this sense, an individual diet could have several dietary factors with anti/pro-inflammatory effects (i.e., fiber, nuts, fruits, and vegetables, among others) [4]. Only two previous studies have reported the relationship between dietary factors and S-Klotho plasma levels [26,27]. The results of these studies demonstrated that nut consumption was positively associated with S-Klotho plasma levels [26], while alcoholic drink consumption was negatively associated with S-Klotho plasma levels in middle-aged adults [27]. However, the relationship between the inflammatory dietary pattern and S-Klotho plasma levels has not been previously investigated.

The DII has been previously validated with several inflammatory markers such as C-reactive protein, interleukin-6, and homocysteine [28,29,30]. Previous studies have suggested that a high DII, which represents a pro-inflammatory diet, may increase the incidence of age-related chronic diseases and all-cause mortality [7,31]. However, to the best of our knowledge, there are no previous studies investigating the association between DII and well-recognized anti-ageing biomarkers such as the S-Klotho protein. Paradoxically, it has been previously demonstrated that S-Klotho plasma levels are increased in critical illness patients (i.e., septic shock) where there is a clear pro-inflammatory state [32,33]. This increment in S-Klotho plasma levels could be the consequence of an attempt to modulate pro-inflammatory pathways [32,33] since S-Klotho protein may downregulate inflammation through the inactivation of NFκB and the suppression of the production of several pro-inflammatory cytokines (i.e., IL-6, IL-8, RANTES, and TNF-α) [20]. Moreover, Gonzalez-Reimers et al. postulated that the development of an inflammatory status produced by high ethanol intake could induce the overexpression of the Klotho gene in order to compensate this pro-inflammatory state [33]. Therefore, it seems plausible that a pro-inflammatory dietary pattern could increase S-Klotho plasma levels to modulate the dietary-mediated inflammatory state.

Some lifestyle factors (i.e., unhealthy dietary habits or sedentariness) have been associated with an increment of the inflammation because they are linked to obesity [34]. In this sense, weight-loss interventions have demonstrated to decrease inflammation markers [35]. In our study, the change in the beta coefficient for the association of DII and S-Klotho plasma levels after adjusting for BMI was 5.572, which supposed an important attenuation of this relationship when any confounder variable is considered. This could be explained because BMI is strongly associated with the inflammatory status [34] and with S-Klotho plasma levels [36]. Therefore, BMI seems to be a strong predictor of several inflammatory and anti-ageing biomarkers [36,37].

We observed that for every 1-unit increment in the DII score, the S-Klotho plasma levels increase 52.223 (raw) and 46.651 pg/mL (adjusted for BMI), respectively. These changes in S-Klotho plasma levels have important clinical implications. In a previous study, S-Klotho plasma levels differed in 45 pg/mL when patients with cardiovascular diseases and healthy individuals were compared (i.e., 626 vs 671 pg/mL respectively) [18]. Furthermore, participants with <575 pg/mL had an increased risk of all-cause mortality compared with those with >763 pg/mL [19]. Therefore, our results have a marked clinical significance considering that changes in DII could influence the risk of cardiovascular disease and all-cause mortality modulating S-Klotho plasma levels [18,19].

The present study suffers from certain limitations. Firstly, it has a cross-sectional design, precluding the establishment of causality. Secondly, the participants were healthy sedentary middle-aged adults (45–65 years old), and we do not know whether these results can be extended to younger/older, unhealthy or physically active populations. Further studies are required to determine whether these results can be found in different populations. Thirdly, it is important to consider the difficulty of an accurate dietary evaluation with possible underreporting or misclassification. Finally, we cannot extrapolate our results to Klotho protein, since we did not know the levels of Klotho protein in different tissues, which could be only measured through a biopsy.

## 5. Conclusions

In conclusion, a pro-inflammatory dietary pattern measured with the DII could influence S-Klotho plasma levels in middle-aged sedentary adults. We observed a slightly positive association between the DII and S-Klotho plasma levels. However, these results should be interpreted with caution because the association was weak and only applied for individuals with specific biological characteristics. Our results may have a potential clinical significance and may support the idea that S-Klotho plasma levels could be increased, aiming to improve a dietary-related pro-inflammatory status in humans. Experimental studies are warranted to determine if a dietary intervention based on an anti-inflammatory diet could influence S-Klotho plasma levels in humans.

## Figures and Tables

**Table 1:** Descriptive parameters.

	All (*N* = 73)	Men (*N* = 35)	Women (*N* = 38)
Age (years)	53.6 (5.2)	54.4 (5.3)	53.0 (5.1)
Energy intake (kcal/day)	2133.9 (688.4)	2067.7 (497.3)	2194.8 (828.9)
DII	−0.19 (1.66)	−0.07 (1.64)	−0.30 (1.93)
BMI (kg/m^2^)	26.7 (3.8)	28.3 (3.6)	25.2 (3.3)
Waist-hip ratio	0.91 (0.08)	0.97 (0.07)	0.86 (0.06)
Use of medication % (*n*)	37.0 (27)	31.4 (11)	42.1 (16)
Systolic blood pressure (mmHg)	127.4 (15.4)	134.2 (13.6)	121.7 (14.6)
Diastolic blood pressure (mmHg)	81.5 (11.7)	85.4 (10.8)	78.2 (11.7)
S-Klotho (pg/mL)	773.7 (366.0)	814.1 (452.2)	737.5 (268.0)

Data are presented as mean and standard deviation. Abbreviations: BMI, Body mass index; DII, dietary inflammatory index.

**Table 2:** Hierarchical regression between the dietary inflammatory index with S-Klotho plasma levels.

	S-Klotho Plasma Levels		
	Β (95% CI)	t	*p*
DII	40.619 (−16.512, 97.749)	1.425	0.160
Energy Intake	0.020 (−0.109, 0.149)	0.311	0.757
Sex	18.719 (−229.935, 267.374)	0.151	0.881
BMI	33.051 (4.675, 61.427)	2.334	0.023
WHiR	−608.476 (−1994.171, 777.218)	−0.880	0.383
Medication use	7.879 (−179.829, 195.5889	0.084	0.933
Systolic blood pressure	3.512 8−7.294, 14.3199	0.651	0.518
Dyastolic blood pressure	−1.188 (−14.393, 12.017)	−0.180	0.858

Non-standarized β coefficient [95% confidence interval], t and *p* value are provided. Abbreviations: DII, dietary inflammatory index; BMI, body mass index; WHiR, waist-hip ratio.
